# RETRACTION: Anticancer Potentials of Root Extract of *Polygala senega* and Its PLGA Nanoparticles‐Encapsulated Form

**DOI:** 10.1155/ecam/9891729

**Published:** 2026-06-29

**Authors:** Evidence-Based Complementary and Alternative Medicine

RETRACTION: S. Paul, S.S. Bhattacharyya, N. Boujedaini, and A.R. Khuda‐Bukhsh, “Anticancer Potentials of Root Extract of *Polygala senega* and Its PLGA Nanoparticles‐Encapsulated Form,” *Evidence-Based Complementary and Alternative Medicine*, vol. 2011 (2011), https://doi.org/10.1155/2011/517204

The above article, published online on 21 September 2010 in Wiley Online Library (https://wileyonlinelibrary.com), has been retracted by John Wiley & Sons Ltd.

The retraction has been agreed following an investigation of the issues raised by a third party on PubPeer [1], which highlighted concerns related to multiple figures within the article. More specifically:

• Figure 1: Multiple unexpectedly similar features were identified within images shown in panels (a) and (b). In addition, the image in panel (a) appears similar to Figure 1 of a previously published article, where it is described as an outcome of a different experiment [2].
• Figure 4a: The images of cells incubated with EEPS after 120, 180, and 240 minutes share an unexpected similarity. Moreover, the images also appear similar to the image of cells treated with 7.5‐fold nano‐coumarin in Figure 3a of another publication by the author group [3].
• Figure 8: The images of the A549 cells appear in Figure 1 of [4], where they describe the staining of the same cell type under a different experimental treatment.

As a result of this investigation, the results of the article are no longer considered reliable. Therefore, the article is retracted.

The authors disagree with this retraction.
